# Unusual Offer

**Published:** 1875-01-01

**Authors:** 


UNUSUAL OFFER. — Dr. E. S. Gaillard, of Louisville, Ky., editor of *The Richmond and Louisville Medical Jour-*

*nal* and the *American Medical Weekly*, makes to the public this unusual and liberal offer: To subscribers to the first journal, twelve handsomely engraved portraits of distinguished European and American physicians; to subscribers to the *Weekly*, one of these portraits in each of the two volumes for 1875. The price of the first journal is $5.00 annually, and that of the last, $2.00 for the same period.

